# PCDH17 increases the sensitivity of colorectal cancer to 5-fluorouracil treatment by inducing apoptosis and autophagic cell death

**DOI:** 10.1038/s41392-019-0087-0

**Published:** 2019-11-29

**Authors:** Shuiping Liu, Haoming Lin, Da Wang, Qiang Li, Hong Luo, Guoxiong Li, Xiaohui Chen, Yongqiang Li, Peng Chen, Bingtao Zhai, Wengang Wang, Ruonan Zhang, Bi Chen, Mingming Zhang, Xuemeng Han, Qiujie Li, Liuxi Chen, Ying Liu, Xiaying Chen, Guohua Li, Yu Xiang, Ting Duan, Jiao Feng, Jianshu Lou, Xingxing Huang, Qin Zhang, Ting Pan, Lili Yan, Ting Jin, Wenzheng Zhang, Lvjia Zhuo, Yitian Sun, Tian Xie, Xinbing Sui

**Affiliations:** 1 Holistic Integrative Pharmacy Institutes and Comprehensive Cancer Diagnosis and Treatment Center, the Affiliated Hospital of Hangzhou Normal University, College of Medicine, Hangzhou Normal University, Hangzhou, Zhejiang China; 2 Key Laboratory of Elemene Class Anti-cancer Chinese Medicine of Zhejiang Province and Engineering Laboratory of Development and Application of Traditional Chinese Medicine from Zhejiang Province, Hangzhou Normal University, Hangzhou, Zhejiang China; 3 Department of Hepatobiliary Pancreatic Surgery, Sun Yat-sen Memorial Hospital, Sun Yat-sen University, Guangzhou, China; 4 Department of Colorectal Surgery, The Second Affiliated Hospital of Zhejiang University School of Medicine, Hangzhou, China; 5 Department of Ultrasound Medicine, The First Affiliated Hospital, School of Medicine, Zhejiang University, Hangzhou, China; 6 Department of Medical Oncology, Sir Run Run Shaw Hospital, Zhejiang University, Hangzhou, China; 7 Jiangsu Key Laboratory for Pharmacology and Safety Evaluation of Chinese Materia Medica, School of Pharmacy, Nanjing University of Chinese Medicine, Nanjing, China

**Keywords:** Oncology, Gastrointestinal cancer

## Abstract

5-Fluorouracil (5-FU) is known as a first-line chemotherapeutic agent against colorectal cancer (CRC), but drug resistance occurs frequently and significantly limits its clinical success. Our previous study showed that the protocadherin 17 (*PCDH17*) gene was frequently methylated and functioned as a tumor suppressor in CRC. However, the relationship between *PCDH17* and 5-FU resistance in CRC remains unclear. Here, we revealed that *PCDH17* was more highly expressed in 5-FU-sensitive CRC tissues than in 5-FU-resistant CRC tissues, and high expression of *PCDH17* was correlated with high BECN1 expression. Moreover, this expression profile contributed to superior prognosis and increased survival in CRC patients. Restoring *PCDH17* expression augmented the 5-FU sensitivity of CRC in vitro and in vivo by promoting apoptosis and autophagic cell death. Furthermore, autophagy played a dominant role in *PCDH17*-induced cell death, as an autophagy inhibitor blocked cell death to a greater extent than the pancaspase inhibitor Z-VAD-FMK. *PCDH17* inhibition by siRNA decreased the autophagy response and 5-FU sensitivity. Mechanistically, we showed that c-Jun NH2-terminal kinase (JNK) activation was a key determinant in *PCDH17*-induced autophagy. The compound SP600125, an inhibitor of JNK, suppressed autophagy and 5-FU-induced cell death in *PCDH17*-reexpressing CRC cells. Taken together, our findings suggest for the first time that *PCDH17* increases the sensitivity of CRC to 5-FU treatment by inducing apoptosis and JNK-dependent autophagic cell death. *PCDH17* may be a potential prognostic marker for predicting 5-FU sensitivity in CRC patients.

## Introduction

Colorectal cancer (CRC) ranks third in morbidity and second in mortality among various malignancies, although much progress has been achieved in CRC therapy in past years.^1^ Currently, the antimetabolite 5-fluorouracil (5-FU) is widely used as chemotherapeutic drug therapy in various solid tumors such as colorectal cancer and gastric cancer. Although 5-FU in combination with other chemotherapeutic agents improves the prognosis of CRC patients, 5-FU resistance occurs frequently and significantly limits its clinical success.^2,3^ Epigenetic and genetic disruptions of tumor suppressor genes (TSGs) are considered to be partly attributed to 5-FU resistance.^4,5^ Therefore, the identification of novel genes that have therapeutic potential as predictive biomarkers of 5-FU chemosensitivity is urgently needed.

The protocadherin 17 (*PCDH17*) gene, a member of the protocadherin family, is frequently methylated and associated with poor prognosis in various cancer types, including esophageal squamous cell carcinoma (ESCC),^6^ urological cancer,^7^ gastric cancer,^8^ and nasopharyngeal cancers.^9^ In a previous study, we demonstrated that *PCDH17* was silenced in most CRC cell lines and that restoring *PCDH17* expression reduced tumor cell growth.^10^ Therefore, *PCDH17* is a potential tumor suppressor in CRC. However, the relationship between *PCDH17* and 5-FU resistance in CRC remains unclear.

Autophagy, an important homeostatic cell recycling system, plays an important role in cellular component degradation and recycling.^11,12^ Chemotherapy agents such as 5-FU may give rise to autophagic responses. This autophagic response can have a prodeath or a prosurvival role and thus contribute to anticancer efficacy or drug resistance, respectively.^13,14^ Therefore, targeting autophagy will provide a potential therapeutic strategy to overcome drug resistance and augment the clinical outcomes of anticancer therapies for patients with cancer. However, to our knowledge, there are still no reports about the role of *PCDH17* in regulating autophagy and 5-FU sensitivity in CRC.

In this study, we first demonstrated that *PCDH17* and BECN1 (Beclin 1, which is autophagy-related) were more highly expressed in 5-FU-sensitive CRC tissues than in 5-FU-resistant CRC tissues and showed a significant positive relationship between high expression of these genes and superior prognosis. Next, we found that *PCDH17* reexpression augmented the 5-FU sensitivity of CRC cells by promoting apoptosis and autophagic cell death. Furthermore, JNK activation was proven to confer 5-FU sensitivity in *PCDH17*-reexpressing cells by inducing death-promoting autophagy. Thus, our findings indicate that PCDH17 plays a critical role in augmenting 5-FU sensitivity by promoting autophagy. *PCDH1*7 will hopefully become a potential predictive biomarker for CRC patients treated with 5-FU.

## Results

### Loss of the *PCDH17* and BECN1 proteins is associated with 5-FU resistance and poor prognosis in CRC patients

**Fig. 1:**
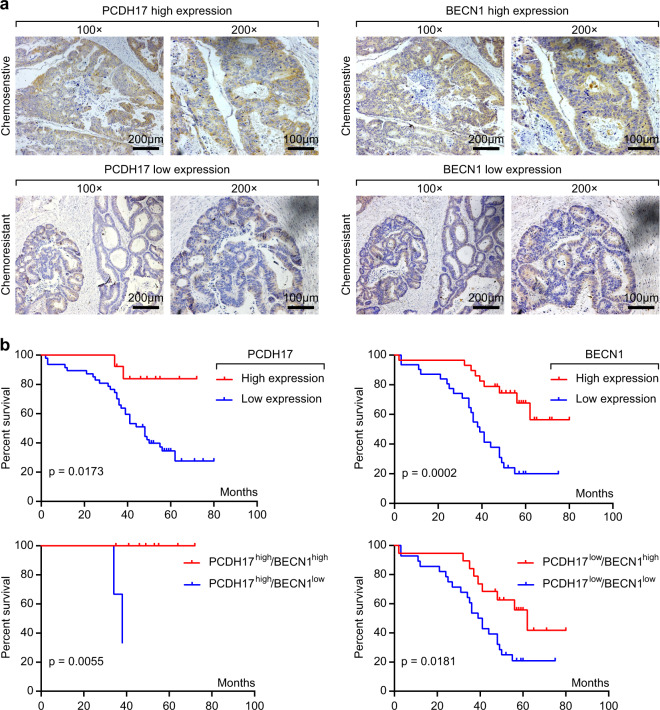
Immunohistochemistry of *PCDH17* and BECN1 expression and patient prognosis and survival analysis. **a** Two serial sections from the same paraffin-embedded block from 60 colorectal cancer patients were used for detection using anti-*PCDH17* and anti-BECN1 antibodies. Representative *PCDH17* and BECN1 staining from a chemosensitive and a chemoresistant sample is shown at ×100 and ×200 magnifications. **b** Kaplan–Meier survival curves for OS in CRC patients with different *PCDH17* and BECN1 protein levels.

In a previous study, we demonstrated that lower protein expression of *PCDH17* was significantly correlated with low T stage, decreased lymph node metastasis, and low tumor stage in CRC patients compared with paired surgical margin tissues. However, the relationship between *PCDH17* expression and the 5-FU sensitivity of CRC patients is still unclear. Therefore, we examined *PCDH17* expression in 21 chemosensitive and 39 chemoresistant CRC tissues by immunohistochemistry (IHC) staining. To explore the potential mechanism of *PCDH17* in cell autophagy, the protein expression of BECN1 was simultaneously detected in the same paraffin-embedded block. The results showed that *PCDH17* and BECN1 were highly expressed in the cytoplasm of cancer cells (Fig. 1a), and their expression was significantly correlated (Table 1, *p* *<* 0.05). In addition, high expression of *PCDH17* and BECN1 was significantly correlated with low levels of lymph node metastasis in both chemosensitive and chemoresistant CRC tissues (Tables 1 and 2, *P* < 0.05). Furthermore, the percentages of cases with high expression of *PCDH17* and BECN1 were 52.4% (11/21 cases) and 81% (17/21 cases), respectively, in chemosensitive CRC tissues but only 7.7% (2/39 cases) and 30.8% (12/39 cases), respectively, in chemoresistant tissues. Kaplan–Meier survival curve analysis indicated that CRC patients with high *PCDH17* expression had significantly better overall survival (OS, *p* = 0.0173) than patients with low *PCDH17* expression. The relationship between BECN1 protein expression and OS was consistent with that of *PCDH17* (Fig. 1b). Moreover, patients with *PCDH17*^high^/BECN1^high^ and *PCDH17*^low^/BECN1^high^ disease had better OS than those with *PCDH17*^high^/BECN1^low^ and *PCDH17*^low^/BECN1^low^ disease, respectively (Fig. 1b). All these data showed that *PCDH17* expression was correlated with sensitivity to 5-FU and autophagy in colorectal cancer patients.

**Table 1:** The association between PCDH17 expression with clinicopathological background and BECN1 experssion.

			PCDH17 immunoreactivity		
		*N*	High (%)	Low (%)	*p* value
Total		60	13 (21.7)	47 (78.3)	
Gender	Male	34	8 (23.5)	26 (76.5)	0.7603
	Female	26	5 (19.2)	21 (80.8)	
Age					
	Median	61			
	≥61	28	5 (15.2)	23 (84.8)	0.9999
	<61	32	8 (29.6)	24 (70.4)	
Histopathological grading					
	Well/moderately	48	10 (20.8)	38 (79.2)	0.7114
	Poorly	12	3 (27.2)	9 (81.8)	
pT categories					
	pT2	9	0 (0)	9 (100)	0.1839
	pT3	51	13 (25.5)	38 (74.5)	
pN categories					
	pN0/1	31	11 (35.5)	20 (64.5)	**0.0109**
	pN2	29	2 (6.9)	27 (93.1)	
Stage					
	II	19	6 (31.6)	13 (68.4)	0.3119
	III	41	7 (17.1)	34 (82.9)	
Chemosensitivity					
	Sensitive	21	11 (52.4)	10 (47.6)	**<0.0001**
	Resistant	39	2 (7.7)	37 (92.3)	
BECN1 expression					
	High	29	10 (34.5)	19 (65.5)	**0.0283**
	Low	31	3 (9.7)	28 (93.5)

**Table 2:** The association between BECN1 expression with clinicopathological background.

			BECN1 immunoreactivity		
		N	High (%)	Low (%)	*p* value
Total		60	29 (48.3)	31 (51.7)	
Gender	Male	34	17 (50.0)	17 (50.0)	0.7997
	Female	26	12 (46.2)	14 (53.8)	
Age					
	Median	61			
	≥61	28	9 (15.2)	19 (84.8)	**0.0227**
	<61	32	20 (29.6)	12 (70.4)	
Histopathological grading					
	Well/moderately	48	23 (20.8)	25 (79.2)	>0.05
	Poorly	12	6 (27.2)	6 (81.8)	
pT categories					
	pT2	9	3 (33.3)	6 (66.7)	0.4743
	pT3	51	26 (51)	25 (49)	
pN categories					
	pN0/1	31	25 (80.6)	6 (19.4)	**<0.001**
	pN2	29	4 (13.8)	25 (86.2)	
Stage					
	II	19	8 (42.1)	11 (57.9)	0.5853
	III	41	21 (51.2)	20 (48.8)	
Chemosensitivity					
	Sensitive	21	17 (81)	4 (19)	**0.0003**
	Resistant	39	12 (30.8)	27 (69.2)	

### Ectopic expression of *PCDH17* induces caspase-dependent apoptosis and autophagy in CRC cells after 5-FU treatment

**Fig. 2:**
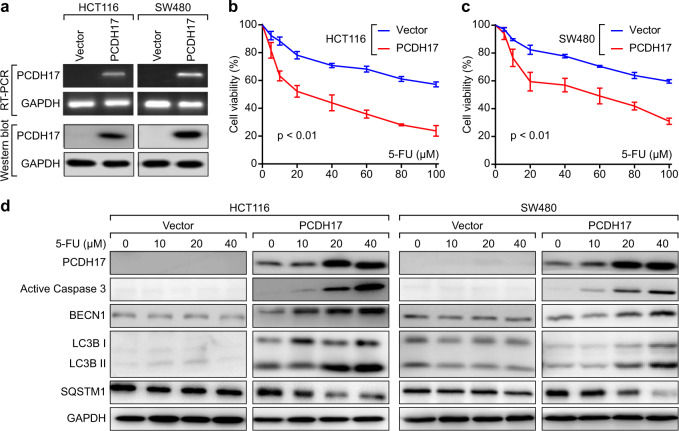
Ectopic expression of *PCDH17*-induced apoptosis and autophagy in HCT116 and SW480 cells. **a** *PCDH17* expression in stably transfected cells as confirmed by RT–PCR and western blot. **b**, **c** Effect of ectopic expression of *PCDH17* on the viability of CRC cells. Cell viability was measured via the CCK-8 assay after 5-FU treatment. Data are presented as the means ± standard deviations (SDs). The experiments were performed in triplicate. **d** Western blot analysis was performed to determine the expression of apoptosis and autophagy proteins in CRC cells transfected with empty vector or *PCDH17* siRNA.

Our previous study showed that methylation mediated the transcriptional silencing of *PCDH17* in CRC cell lines, including HCT116 and SW480 cells. We also established 5-FU-resistant HCT116 cells. However, in agreement with the results in 5-FU-sensitive HCT116 cells, we did not observe PCDH17 expression in HCT116/5-FU-resistant cells (Supplemental Fig. 1). In this study, we detected the effects of ectopic *PCDH17* expression on CRC cell growth. The forced expression of *PCDH17* in HCT116 and SW480 cells was confirmed by RT-PCR and western blot (Fig. 2a). We next investigated whether tumor cell growth was inhibited by ectopic *PCDH17* expression. The CCK-8 assay results showed that *PCDH17*-transfected cells exhibited significantly lower viability than controls after treatment with various concentrations of 5-FU for 24 h (Fig. 2b, c). We further studied the effect of *PCDH17* expression on apoptosis and autophagy with 5-FU treatment. As shown in Fig. 2d, an accumulation of *PCDH17* protein was detected in CRC cells treated with different concentrations of 5-FU. The accumulated *PCDH17* caused apoptotic cell death and triggered autophagy in a 5-FU dose-dependent manner, indicating that apoptosis and autophagy were involved in the effect of *PCDH17* on CRC cell viability. Therefore, we used 20 μM 5-FU in *PCDH17*-transfected HCT116 and SW480 cells in subsequent experiments.

### Restoration of *PCDH17* expression activates autophagic cell death in CRC cells

**Fig. 3:**
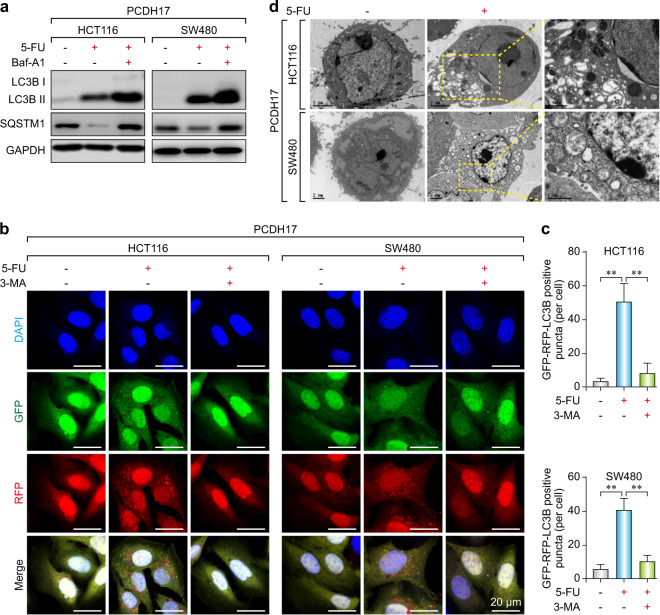
*PCDH17* increases autophagosome formation and autophagic flux in CRC cells. **a** *PCDH17*-transfected HCT116 and SW480 cells were treated with 20 μM 5-FU with or without 10 μM BafA1 for 24 h, and the protein levels of LC3B and SQSTM1 were assessed by western blotting. **b** HCT116/*PCDH17* and SW480/*PCDH17* cells were transfected with the GFP-RFP-LC3 plasmid overnight and transferred onto coverslips. After exposure to 20 μM 5-FU with or without 3-MA for 24 h, representative images of LC3-II-positive puncta were obtained with a confocal fluorescence microscope. **c** The quantitative analyses of the number of fluorescent puncta are shown. The experiments were performed in triplicate. **p < 0.01. **d** Electron microscopy shows the ultrastructures of autophagosome and autolysosome vesicles in these cells. The experiments were performed in triplicate. Bar = 2 μm.

It is known that autophagy is one of the conserved protein degradation processes, and it can lead to resistance to chemotherapy treatment through a cell survival or cell death mechanism, depending on the cellular status. To determine the effect of *PCDH17* on the autophagy induced by 5-FU, we investigated the level of LC3-II, the number of autophagic puncta and the formation of autophagosomes in HCT116 and SW480 cells. Western blotting results revealed that *PCDH17* increased the turnover of the autophagic marker LC3B-II and SQSTM1 (sequestosome 1) degradation (Fig. 3a). Moreover, LC3B-II levels were further increased and SQSTM1 was increased after BafA1 (bafilomycin A_1_) treatment, indicating that 5-FU induced the autophagic flux of *PCDH17*-transfected CRC cells. In accordance with these results, the number of GFP-RFP-LC3-II-positive puncta in *PCDH17*-transfected CRC cells was significantly higher than that in the control cells after 24 h exposure to 20 μM 5-FU, whereas little signal was observed after treatment with the autophagy inhibitor 3-MA (3-methyladenine) (Fig. 3b, c). The TEM results also indicated that there were more autophagosomes and autolysosomes in the cytoplasm of *PCDH17*-transfected cells than in that of control cells (Fig. 3d).

**Fig. 4:**
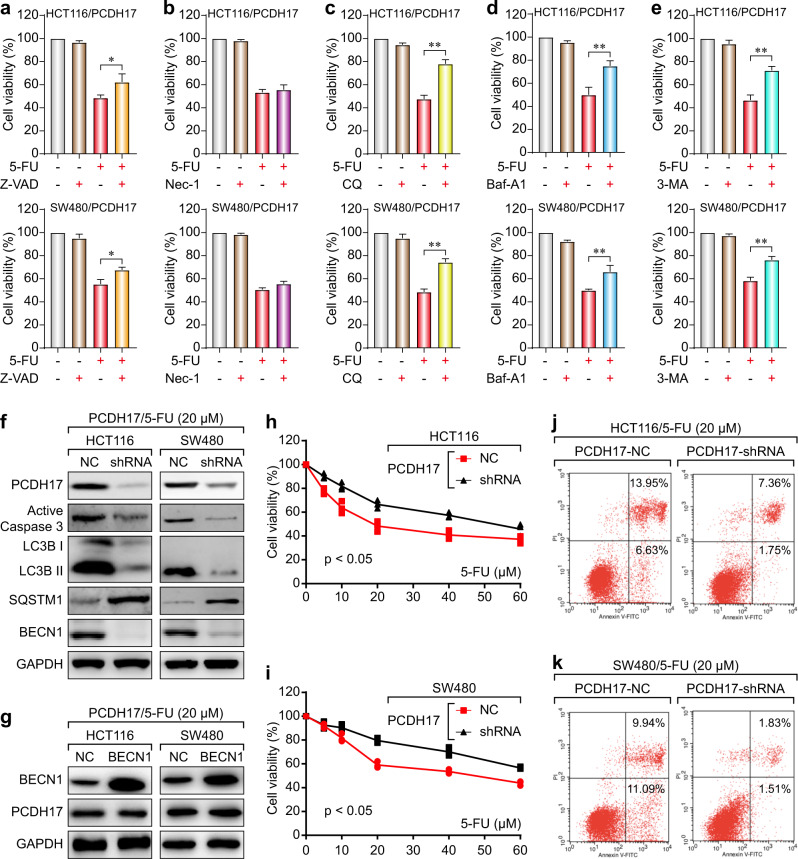
Autophagy contributes to *PCDH17*-induced growth inhibition in CRC cells, and *PCDH17* regulates autophagy and 5-FU sensitivity. **a** HCT116/*PCDH17* and SW480/*PCDH17* cells were treated with 5-FU with or without the pancaspase inhibitor Z-VAD-FMK for 24 h, and cell viability was assayed. The experiments were performed in triplicate. *p < 0.05. **b** HCT116/*PCDH17* and SW480/*PCDH17* cells were treated with 5-FU with or without the necroptosis inhibitor necrostatin-1 for 24 h, and cell viability was assayed. The experiments were performed in triplicate. **c** HCT116/*PCDH17* and SW480/*PCDH17* cells were treated with 5-FU with or without the autophagy inhibitor CQ (20 μM) for 24 h, and cell viability was assayed. The experiments were performed in triplicate. **p < 0.01. **d** HCT116/*PCDH17* and SW480/*PCDH17* cells were treated with 5-FU with or without the autophagy inhibitor BafA1 (0.05 nM) for 24 h, and cell viability was assayed. The experiments were performed in triplicate. **p < 0.01. **e** HCT116/*PCDH17* and SW480/*PCDH17* c**e**lls were treated with 5-FU with or without the autophagy inhibitor 3-MA (5 μM) for 24 h, and cell viability was assayed. The experiments were performed in triplicate. **p < 0.01. **f** HCT116/*PCDH17* and SW480/*PCDH17* cells were transfected with *PCDH17*-specific shRNA, and then *PCDH17*, caspase-3, BECN1 and LC3B levels were assessed by western blotting. **g** HCT116/*PCDH17* and SW480/*PCDH17* cells were transfected with BECN1 plasmid, and then BECN1 and *PCDH17* levels were assessed by western blotting. **h**, **i** HCT116/*PCDH17* and SW480/*PCDH17* cells were transfected with PCDH17-specific shRNA, and then the cells were exposed to 5-FU at various final concentrations for 24 h. Cell viability was measured by CCK-8 assay after 48 h. Data are presented as the means ± standard deviations (SDs). The experiments were performed in triplicate. **j**, **k** HCT116/*PCDH17* and SW480/*PCDH17* cells were transfected with *PCDH17*-specific shRNA, and then the cells were exposed to 5-FU at various final concentrations for 24 h. Cell viability was measured by an Annexin V–FITC dual staining assay followed by flow cytometry after 48 h. Data are presented as the means ± standard deviations (SDs). The experiments were performed in triplicate.

To investigate whether *PCDH17*-triggered autophagy plays a prosurvival or prodeath role, *PCDH17*-transfected CRC cells were exposed to 5-FU with or without different inhibitors. The 5-FU treatment combined with Z-VAD-FMK (a pancaspase inhibitor) or the autophagy inhibitor chloroquine (CQ), BafA1 and 3-MA, but not with the necroptosis inhibitor necrostatin-1, prevented the *PCDH17*-induced cell growth inhibition (Fig. 4a–e). Furthermore, autophagy played a dominant role in *PCDH17*-induced cell death, since the autophagy inhibitors blocked cell death to a greater extent than the pancaspase inhibitor Z-VAD-FMK (Fig. 4a, c–e). These results strongly indicate that *PCDH17*-induced apoptosis and autophagy collectively trigger cell death. Additionally, autophagy serves as the predominant method by which *PCDH17* induces cell death.

### *PCDH17* regulates autophagy and apoptosis and augments 5-FU sensitivity

Next, we tested the effect of *PCDH17* knockdown on autophagy and 5-FU sensitivity by using short hairpin RNA (shRNA) to knockdown *PCDH17* expression in *PCDH17*-transfected CRC cells. Both HCT116/*PCDH17* and SW480/*PCDH17* cells were transfected with *PCDH17*-specific shRNA. Decreased expression of *PCDH17* was confirmed by western blotting (Fig. 4f). We found that *PCDH17* knockdown by specific shRNA significantly inhibited LC3B-II turnover and the expression of BECN1 and active caspase-3 (Fig. 4f); however, BECN1 overexpression did not affect *PCDH17* expression (Fig. 4g), indicating that *PCDH17* can modulate BECN1 and apoptosis.

We then assessed *PCDH17* regulation of 5-FU sensitivity in HCT116 and SW480 cells. The CCK-8 assay showed that the cytotoxic effect of 5-FU was significantly inhibited in a dose-dependent manner when *PCDH17* was knocked down compared with the controls (Fig. 4h, i). To quantify the cell viability, we performed an Annexin V-FITC dual staining assay and then flow cytometry. In line with the above findings, flow cytometry results also showed that *PCDH17* knockdown significantly decreased 5-FU sensitivity in HCT116/*PCDH17* and SW480/*PCDH17* cells (Fig. 4j, k). Taken together, these data suggested that *PCDH17* could modulate autophagy and augment the 5-FU sensitivity of CRC cells.

### Restoration of *PCDH17* expression activates autophagic cell death to augment the 5-FU sensitivity of HCT116 cells in vivo

To investigate whether *PCDH17* can sensitize cells to the anticancer effects of 5-FU in vivo, xenograft tumor models of HCT116/PCDH17 cells were generated. When the size of tumor xenografts reached 100 mm^3^, the mice were randomly divided into 4 groups: the control group, 5-FU group (40 mg/kg intraperitoneal injection every 2 days for 28 days), 3-MA group (20 mg/kg intraperitoneal injection every 2 days for 28 days), and 5-FU plus 3-MA group.

**Fig. 5:**
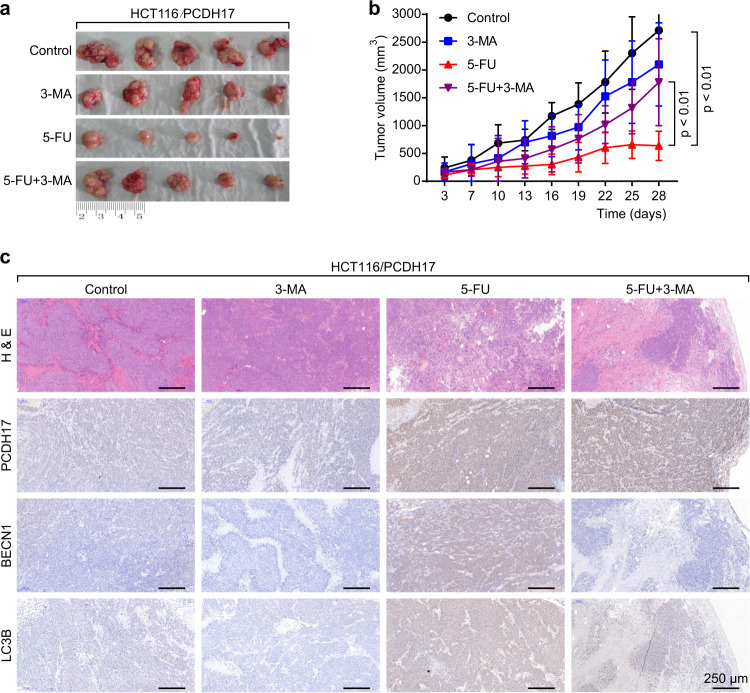
*PCDH17* regulates autophagy and augments 5-FU sensitivity in vivo. **a** Representative image of *PCDH17* xenograft tumors. **b** Tumor volume in each group. Data are expressed as the means ± standard deviations (SDs). **c** H&E and immunohistochemical staining of tumor specimens. Scale bars = 250 μm.

As shown in Fig. 5a, b, not 3-MA but 5-FU alone (*p* *<* 0.01) exhibited a significant effect on the growth of HCT116/*PCDH17* CRC cells. In accordance with the in vitro results, the tumor size of the 5-FU plus 3-MA group was significantly larger than that of the 5-FU group (*p* *<* 0.01), indicating that *PCDH17* can induce prodeath autophagy in vivo when CRC cells are treated with 5-FU. Furthermore, no significant hepatic toxicity or weight loss was observed in the 5-FU group or the 5-FU plus 3-MA group (data not shown).

Next, we examined the protein expression of *PCDH17*, BECN1 and LC3B by immunohistochemical staining. As shown in Fig. 5c, 5-FU treatment resulted in an upregulation of *PCDH17* and autophagy in HCT116/*PCDH17* CRC cells.

Taken together, our data indicated that *PCDH17* sensitized CRC cells to the anticancer effects of 5-FU in vivo by inducing autophagic cell death. By inhibiting autophagy, 3-MA potently attenuated the antitumor activity of 5-FU in vivo.

### *PCDH17* activates autophagy via the c-Jun N-terminal kinase (JNK) pathway

**Fig. 6:**
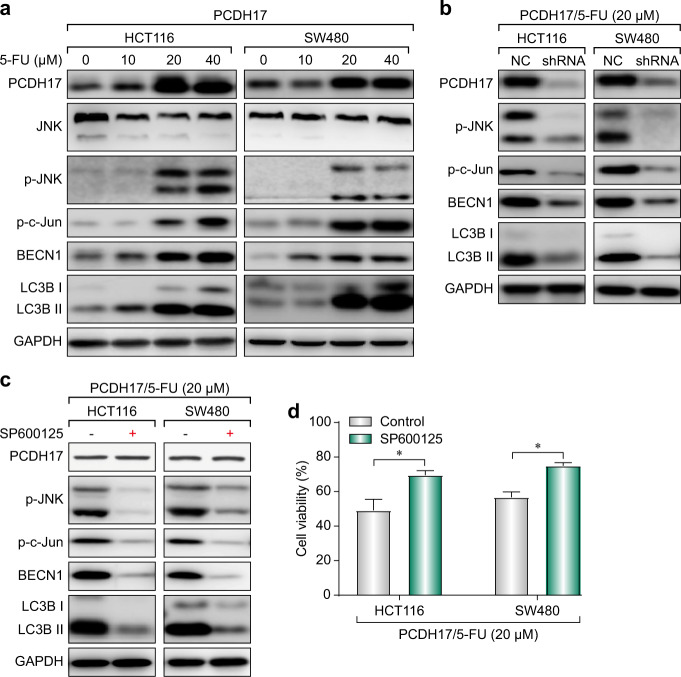
*PCDH17* meditates JNK activation to augment the cytotoxic effect of 5-FU in CRC cells by inducing prodeath autophagy. **a** HCT116/*PCDH17* and SW480/*PCDH17* cells were treated with varying concentrations of 5-FU for 24 h, and then *PCDH17*, JNK signaling, and LC3B were assessed by western blotting. **b** HCT116/*PCDH17* and SW480/*PCDH17* cells were transfected with PCDH17-specific shRNA, and then *PCDH17*, JNK signaling, BECN1 and LC3B were assessed by western blotting. **c** HCT116/*PCDH17* and SW480/*PCDH17* cells were treated with the combination of 5-FU and SP600125, and then *PCDH17*, JNK signaling, BECN1 and LC3B were assessed by western blotting. **d** HCT116/*PCDH17* and SW480/*PCDH17* cells were treated with the combination of 5-FU and SP600125. Cell viability was measured by CCK-8 assay. Data are presented as the means ± standard deviations (SDs). The experiments were performed in triplicate. *p < 0.05.

c-Jun N-terminal kinase (JNK) plays a key role in the sensitivity to and outcome of anticancer therapies. JNK activation can mediate BECN1 expression to induce autophagic cell death in response to chemotherapeutic agents.^15^ Because we observed that autophagic cell death was involved in the effect of *PCDH17* on CRC cell viability, we next detected the protein levels of phosphorylated-JNK (p-JNK) to determine whether the JNK pathway is activated in *PCDH17*-transfected cells when exposed to 5-FU treatment. As shown in Fig. 6a, we found that *PCDH17* was upregulated in 5-FU-treated HCT116/*PCDH17* and SW480/*PCDH17* cells in a dose-dependent manner, which might be attributed to endogenous *PCDH17* expression (Supplemental Fig. 2). JNK activation was observed along with *PCDH17* upregulation, suggesting that JNK may be activated in these cells exposed to 5-FU. For further confirmation, we preformed western blotting and found that phosphorylated c-Jun was significantly induced in HCT116/*PCDH17* and SW480/*PCDH17* cells in a 5-FU dose-dependent manner. Moreover, increases in BECN1 expression and the LC3-II/I ratio were observed when HCT116/*PCDH17* and SW480/*PCDH17* cells were treated with 5-FU (Fig. 6a).

Next, we used shRNA to knockdown *PCDH17* expression in *PCDH17*-transfected CRC cells and studied the effect of such *PCDH17* inhibition on JNK activation and autophagy. Western blotting was used to confirm the reduced expression of *PCDH17* (Fig. 6b). *PCDH17* knockdown by specific shRNA significantly inhibited JNK activation and LC3B-II turnover (Fig. 6b), indicating that *PCDH17* can modulate JNK activation and the induction of autophagy.

To determine whether JNK activation may be correlated with autophagy induction in HCT116/*PCDH17* and SW480/*PCDH17* cells in response to 5-FU, we specifically inhibited JNK activation with the JNK inhibitor SP600125 (10 µM). As shown in Fig. 6c, inhibiting JNK in *PCDH17*-transfected CRC cells significantly decreased the level of phosphorylated c-Jun and the ratio of LC3-II/I but had no effect on *PCDH17* expression. Moreover, inhibition of autophagy by JNK knockdown attenuated the cytotoxicity activity of 5-FU (Fig. 6d). These results demonstrated that *PCDH17*-induced autophagic cell death by activating the JNK signaling pathway.

## Discussion

CRC is the third most common malignancy worldwide and associated with high-mortality. To date, 5-FU-based chemotherapy remains widely used in the clinical treatment of CRC patients, but resistance to 5-FU is a major drawback in its clinical use.^16^ Thus, overcoming 5-FU resistance or increasing 5-FU treatment efficacy is urgently needed.

*PCDH17*, a member of the cadherin superfamily, functions as a potential tumor suppressor and is found to be transcriptionally silenced in various human cancer types due to molecular mechanisms such as deletion, mutation, and promoter methylation.^17,18^ Our previous study reported that *PCDH17* is frequently methylated and inactivated in gastric and colorectal cancers, in which *PCDH17* functions as a tumor suppresser by inducing apoptosis and autophagy.^10^ However, no further detailed analysis of the biological roles of *PCDH17* in autophagy and the sensitivity of CRC cells to 5-FU has been conducted to date.

In this study, the protein expression of *PCDH17* and BECN1 was detected in CRC tissues by IHC. We first revealed that the expression of *PCDH17* and BECN1 was significantly upregulated in chemosensitive tissues compared with chemoresistant tissues. Moreover, the clinical analysis results indicated that high expression of *PCDH17* and BECN1 was significantly correlated with better overall survival and higher 5-FU sensitivity than were seen with low expression of *PCDH17* and BECN1. Next, we further investigated the effect of 5-FU on *PCDH17* expression and found an accumulation of *PCDH17* protein in CRC cells treated with different concentrations of 5-FU. The accumulated *PCDH17* caused apoptosis and autophagy in a 5-FU dose-dependent manner. Moreover, autophagy serves as the predominant method by which *PCDH17* induces cell death. *PCDH17* knockdown by shRNA or autophagy inhibition significantly decreased the sensitivity to 5-FU in *PCDH17*-transfected CRC cells. Taken together, these data suggest that *PCDH17* can augment the 5-FU sensitivity of CRC cells by modulating autophagy.

The JNK pathway plays a key role in cancer genesis and development, including processes such as DNA repair, cell proliferation, apoptosis, metabolism and motility. When cells encounter genotoxic stress, JNK is also involved in autophagy induction. Thus, we postulated that *PCDH17* could activate the JNK pathway and mediate autophagic cell death in CRC cells. To clarify this hypothesis, we focused on the correlation between JNK pathway activation and *PCDH17*-induced autophagy. Here, we demonstrated that *PCDH17* was upregulated in a dose-dependent manner, which was observed along with JNK activation, suggesting that JNK could be activated in these cells after 5-FU treatment. On the other hand, PCDH17 knockdown and the JNK-specific inhibitor SP600125 inhibited autophagy induction and attenuated the cytotoxicity activity of 5-FU, but JNK knockdown had no effect on *PCDH17* expression.

Taken together, the present study showed that CRC patients with high expression of *PCDH17* and BECN1 had a better prognosis than CRC patients with low expression of *PCDH17* and BECN1. 5-FU induces *PCDH17* upregulation and autophagic cell death in CRC cells, and the ectopic expression of *PCDH17* augments the 5-FU sensitivity of CRC cells by promoting JNK-dependent autophagic cell death in vitro and in vivo. Our findings first suggest that *PCDH17* increases the 5-FU sensitivity of CRC cells by inducing JNK-dependent autophagic cell death, which might support the clinical potential of *PCDH17* for predicting 5-FU sensitivity in CRC patients.

## Methods

### Cell lines and reagents

The human colorectal cancer cell lines HCT116 and SW480 were obtained from the ATCC (LGC Standards SLU, Barcelona, Spain). They were cultured in McCoy’s 5 A or Dulbecco’s modified Eagle’s medium (DMEM; Gibco BRL, Rockville, MD, USA) containing 10% fetal bovine serum (FBS), 100 units/mL penicillin, 100 µg/mL streptomycin (Invitrogen), and 2 mmol/L L-glutamine in a humidified atmosphere of 5% carbon dioxide in air at 37 °C. The fetal bovine serum was obtained from Corille (184590, Australia). 5-FU was obtained from Jinyao Amino Acid Co., Ltd. (Tianjin, China).

BafA1 (B1793), 3-methyladenine (M9281), chloroquine (C6628), the pancaspase inhibitor Z-VAD-FMK (C2105), necrostatin-1 (N9037) and SP600125 (S5567) were purchased from Sigma Aldrich. Anti-LC-3B (#3868), anti-p62 (#8025), anti-cleaved caspase-3 (#9661), anti-SQSTM1/p62 (#5114), anti-BECN1 (#3495), anti-SAPK/JNK (#9252), anti-phospho-SAPK/JNK (Thr183/Tyr185) (81E11) (#4668), anti-phospho-c-Jun (Ser63) (54B3) (#2361), and anti-GAPDH (#5174) antibodies were purchased from Cell Signaling Technology (CST). Anti-PCDH17 (HPA026817) for IHC was obtained from Sigma Aldrich. Anti-PCDH17 (ab128815) for WB was obtained from Abcam. The PCDH17 plasmid (NM_001040429) was purchased from OriGene.

### Patient-specimen selection and immunohistochemistry

IHC was performed with 60 formalin-fixed and paraffin-embedded tissue samples obtained from CRC patients diagnosed from January 2008 to October 2010. All patients volunteered to participate in the initial surgery and then received chemotherapy based on 5-FU. The follow-up deadline was June 30, 2018. Patients who developed disease during the initial treatment or relapsed within 6 months after completing the initial treatment were termed 5-FU-resistant. Patients who relapsed after 6 months or had no recurrence were termed 5-FU-sensitive.

Briefly, according to the instructions of the ChemMate EnVision test kit (Dako, Carpinteria, California, USA), the sections were incubated with the respective primary antibody overnight at 4 °C. The ChemMate EnVision/HRP, Rabbit/Mouse (ENV) reagent was applied to these sections, and then the ChemMate DAB chromogen was used. The slides were then slightly counterstained with hematoxylin.

The expression of PCDH17 and BECN1 proteins was scored according to the intensity of membrane and cytoplasmic staining using a four-point system: 0, negative; 1, weak; 2, moderate; and 3, strong. To investigate the correlation between PCDH17 and BECN1 expression and clinicopathological features, CRC patients were divided into two groups: low expression (0 and 1, –) or high expression (2 and 3, + ). Immunostaining was independently scored by two investigators who did not know the patient’s clinical information in separate cases.

### PCDH17-expressing plasmids and transfection

The pCMV6 Entry-PCDH17 plasmid or empty vector (pCMV6 Entry-mock; OriGene, MD, USA) was transfected into the colorectal cell lines HCT116 and SW480 with the MegaTran 1.0 transfection reagent (OriGene), followed by selection of stable clones expressing PCDH17 or the empty vector for further study.

### Cell Viability and Apoptosis Measurement

Cell viability was assessed using the Cell Counting Kit-8 (CCK-8) (LJ621, DOJINDO, JAPAN) following the product’s introduction. Cells were seeded at a density of 5,000 cells per well in 96-well flat bottom microtiter plates. After 24 h, 5-FU was added at different times at the indicated concentrations. The absorbance was measured at 450 nm on a microplate reader (Synergy HT, Bio-Tek, USA). Apoptosis was detected using a Pharmingen Annexin V-FITC Apoptosis Detection Kit I (BD, USA) following the manufacturer’s instructions. Briefly, cells were seeded at a density of 2 × 10^6^ cells per 6 cm culture dish. After attachment overnight, cells were washed twice with PBS and cultured in medium with 20 µM 5-FU for 24 h. All cells were collected and resuspended in ice-cold 1 × binding buffer. Approximately 1 × 10^5^ cells were then stained with 5 μL of Annexin V-FITC and 5 μL of PI for 15 min and analyzed using a FACS Calibur flow cytometer (Beckman Coulter, CytoFLEX S, USA).

### Immunofluorescent confocal laser microscopy for LC3 and lysosome colocation

HCT116/*PCDH17* and SW480/*PCDH17* cells were transfected overnight with a GFP-RFP-LC3 plasmid and transferred to coverslips. After adhering to the coverslips, the cells were treated with 10 nM 5-FU plus 3-MA or not treated for 24 h. The nuclei were stained with 4′,6-diamidino-2-phenylindole (DAPI, Sigma-Aldrich, D9542), fixed with 4% paraformaldehyde (Sigma-Aldrich, 158127), and permeated with 0.5% Triton X-100 (Solarbio, T8200-100) in PBS (Corning, R21-040-CV). Images were taken with a spinning disk confocal fluorescence microscope (the system consists of the CSU-X1 rotating disk produced by Yokogawa, an IX81 microscope from Olympus and an IXON3 CCD from Andor) with a magnification of 600.

### Western Blot Analysis

Cells were collected from cultured dishes and lysed in lysis buffer. The main components of the lysis buffer were 20 mM Tris-HCl pH 7.6, 1 mM EDTA, 140 mM NaCl, 1% NP-40, 1% aprotinin, 1 mM phenylmethylsulfonyl fluoride (PMSF), and 1 mM sodium vanadate. Protein concentration was determined using a BCA Protein Assay Kit (Pierce). Protein samples (40 μg of protein per cell line) were separated on a 5 to 20% Tris-Tricine Ready Gel (Bio-Rad) by SDS-PAGE for nitrocellulose membrane blotting. The blotted membrane was blocked with 5% skim milk for 1 h at room temperature and incubated overnight at 4 °C with primary antibody. The immunoreactive band was observed by enhanced chemiluminescence using horseradish peroxidase-conjugated IgG secondary antibodies. Band density was measured by densitometry, quantified using NIH image 1.62 gel map macros, and normalized to the specified sample in the same membrane.

### RNA interference

PCDH17 human shRNA (OriGene, TR310584) was transfected with Lipofectamine RNAi Max (Invitrogen, 13778150) following the manufacturer’s instructions.

### Transmission electron microscopy

The treated cells were washed and fixed in 2.5% glutaraldehyde (Sigma-Aldrich, G5882) for 30 min. The samples were treated with 1.5% osmium tetroxide, dehydrated with acetone and embedded in Durcupan resin. Thin sections were poststained with lead citrate and examined with a 60 kV TECNAI 10 electron microscope (Philips, Holland).

### In vivo subcutaneous tumor model

All in vivo experimental protocols were approved by the Animal Protection Committee of Hangzhou Normal University. Viable HCT116/PCDH17 cells (1 × 10^7^) were subcutaneously injected into the right dorsal flank of 6-week-old female BALB/c nude mice (five mice per group). Tumor volume was measured every 2 days for 4 weeks and calculated by the following formula: (short diameter)^2^ × (long diameter)/2.

### Statistical analyses

Data are expressed as the means ± SD of three independent experiments. Prism 7.0c GraphPad software was used for statistical analysis. Significant differences between groups were analyzed by Student’s *t* test, and a *p* value < 0.05 was considered statistically significant.

## Supplementary information

Supplemental material

## References

[1] Bray F (2018). Global cancer statistics 2018: GLOBOCAN estimates of incidence and mortality worldwide for 36 cancers in 185 countries. CA Cancer J. Clin..

[2] Longley DB, Harkin DP, Johnston PG (2003). 5-fluorouracil: mechanisms of action and clinical strategies. Nat. Rev. Cancer.

[3] Wasserman I (2019). SMAD4 loss in colorectal cancer patients correlates with recurrence, loss of immune infiltrate, and chemoresistance. Clin. Cancer Res..

[4] Ou J (2019). ABHD5 blunts the sensitivity of colorectal cancer to fluorouracil via promoting autophagic uracil yield. Nat. Commun..

[5] Henricks LM, Opdam FL, Beijnen JH, Cats A, Schellens JHM (2017). DPYD genotype-guided dose individualization to improve patient safety of fluoropyrimidine therapy: call for a drug label update. Ann. Oncol..

[6] Haruki S (2010). Frequent silencing of protocadherin 17, a candidate tumour suppressor for esophageal squamous cell carcinoma. Carcinogenesis.

[7] Costa VL (2011). TCF21 and PCDH17 methylation: An innovative panel of biomarkers for a simultaneous detection of urological cancers. Epigenetics.

[8] Yang Y, Liu J, Li X, Li JC (2012). PCDH17 gene promoter demethylation and cell cycle arrest by genistein in gastric cancer. Histol. Histopathol..

[9] He, Y. et al. Protocadherin 17 is a tumor suppressor and is frequently methylated in nasopharyngeal carcinoma. *Cancer Manag. Res.* **11**, 1601–1613 (2019). 10.2147/CMAR.S191102 PMC6388982 30863170

[10] Hu X (2013). Protocadherin 17 acts as a tumour suppressor inducing tumour cell apoptosis and autophagy, and is frequently methylated in gastric and colorectal cancers. J. Pathol..

[11] Nassour J (2019). Autophagic cell death restricts chromosomal instability during replicative crisis. Nature.

[12] Levine B, Kroemer G (2019). Biological functions of autophagy genes: a disease perspective. Cell.

[13] Levy JMM, Towers CG, Thorburn A (2017). Targeting autophagy in cancer. Nat. Rev. Cancer.

[14] Sui X (2013). Autophagy and chemotherapy resistance: a promising therapeutic target for cancer treatment. Cell Death Dis..

[15] Gaballah HH, Gaber RA, Mohamed DA (2017). Apigenin potentiates the antitumor activity of 5-FU on solid Ehrlich carcinoma: Crosstalk between apoptotic and JNK-mediated autophagic cell death platforms. Toxicol. Appl Pharm..

[16] Kaehler C, Isensee J, Hucho T, Lehrach H, Krobitsch S (2014). 5-Fluorouracil affects assembly of stress granules based on RNA incorporation. Nucleic Acids Res..

[17] Baranova I (2018). Aberrant methylation of PCDH17 gene in high-grade serous ovarian carcinoma. Cancer Biomark..

[18] Byzia E (2018). Recurrent transcriptional loss of the PCDH17 tumor suppressor in laryngeal squamous cell carcinoma is partially mediated by aberrant promoter DNA methylation. Mol. Carcinog..

