# Supplementary material for: PCDH17 increases the sensitivity of colorectal cancer to 5-fluorouracil treatment by inducing apoptosis and autophagic cell death
Source: Signal Transduct Target Ther. 2019 Nov 29;4:53. doi: 10.1038/s41392-019-0087-0 (PMC6882894; doi:10.1038/s41392-019-0087-0)
Supplement: Supplementary file 1 — Supplemental material [file 41392_2019_87_MOESM1_ESM.docx]

Supplementary Materials for

PCDH17 increases the sensitivity of colorectal cancer to 5-Fluorouracil treatment by inducing apoptosis and autophagic cell death

Shuiping Liu^1, 2,†^, Haoming Lin^3,†^, Da Wang^4,†^, Qiang Li^5^, Hong Luo^1^, Guoxiong Li^1^, Xiaohui Chen^1^, Yongqiang Li^1^, Peng Chen^1^, Bingtao Zhai^1^, Wengang Wang^1^, Ruonan Zhang^1^, Bi Chen^1^, Mingming Zhang^1^, Xuemeng Han^1^, Qiujie Li^1^, Liuxi Chen^1^, Ying Liu^6^, Xiaying Chen^1^, Guohua Li^1^, Yu Xiang^1^, Ting Duan^1^, Jiao Feng^1^, Jianshu Lou^1^, Xingxing Huang^1^, Qin Zhang^1^, Ting Pan^1^, Lili Yan^1^, Ting Jin^1^, Wenzheng Zhang^1^, Lvjia Zhuo^1^, Tian Xie^1,^ ^2, *^, Xinbing Sui^1,^ ^2, 7, *^

†These authors contributed equally to this work

Correspondence to: Tian Xie, Email: xbs@hznu.edu.cn or Xinbing Sui, Email: hzzju@zju.edu.cn

Figure. S1


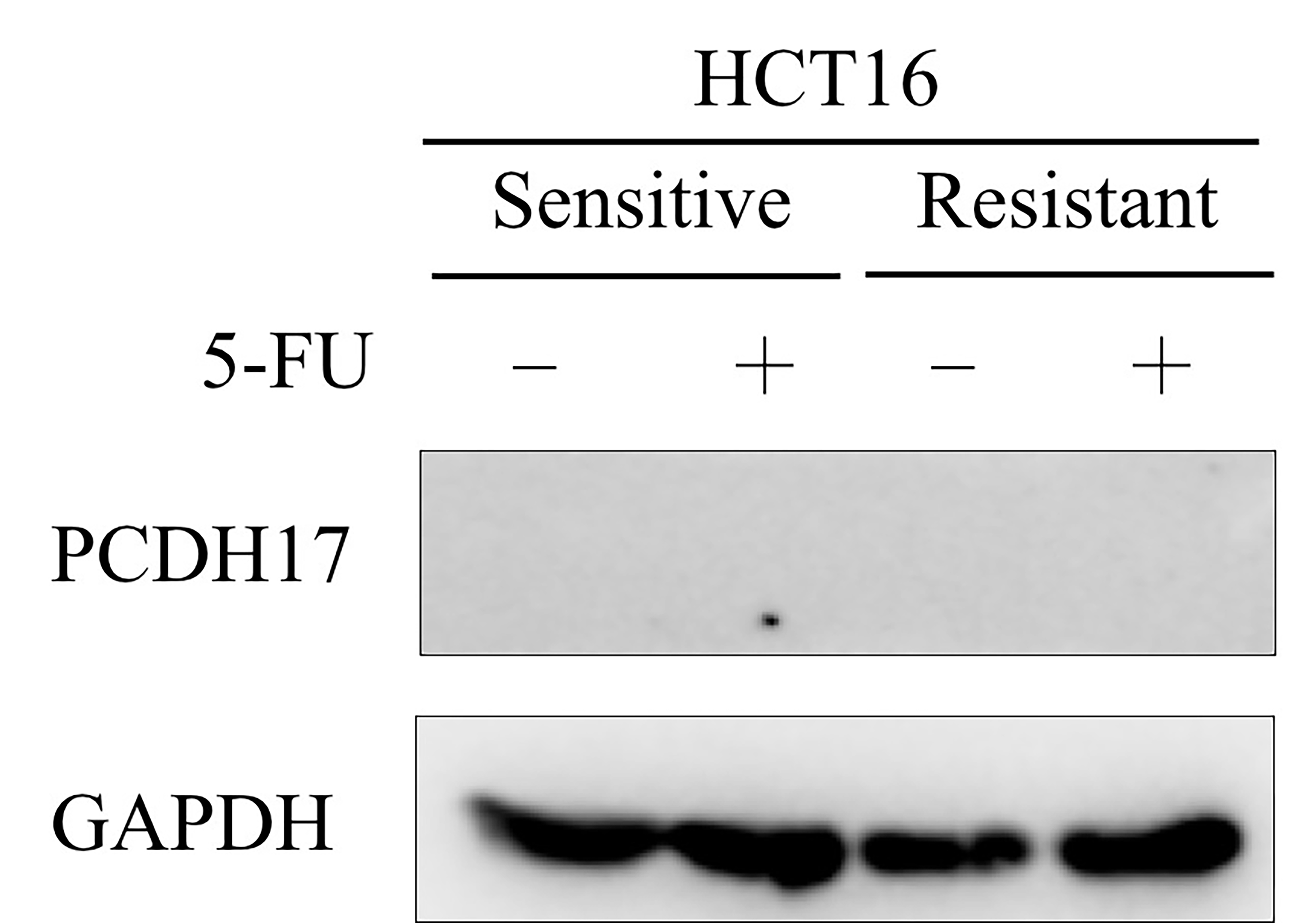


**Figure S1. PCDH17 expression in 5-FU sensitive and resistant HCT116 cells was determined by western blot.** 5-FU sensitive and established 5-FU resistant HCT116 cell lines were used. 24 hours after treating cells with or without 20 μM 5-FU, cells were harvested from cultured dishes and were lysed in a RIPA lysis buffer supplemented with inhibitors of proteases. Cell lysates were separated on SDS-PAGE and determined the expression of PCDH17 by western bolt. GAPDH was used as internal control.

**Figure. S2**


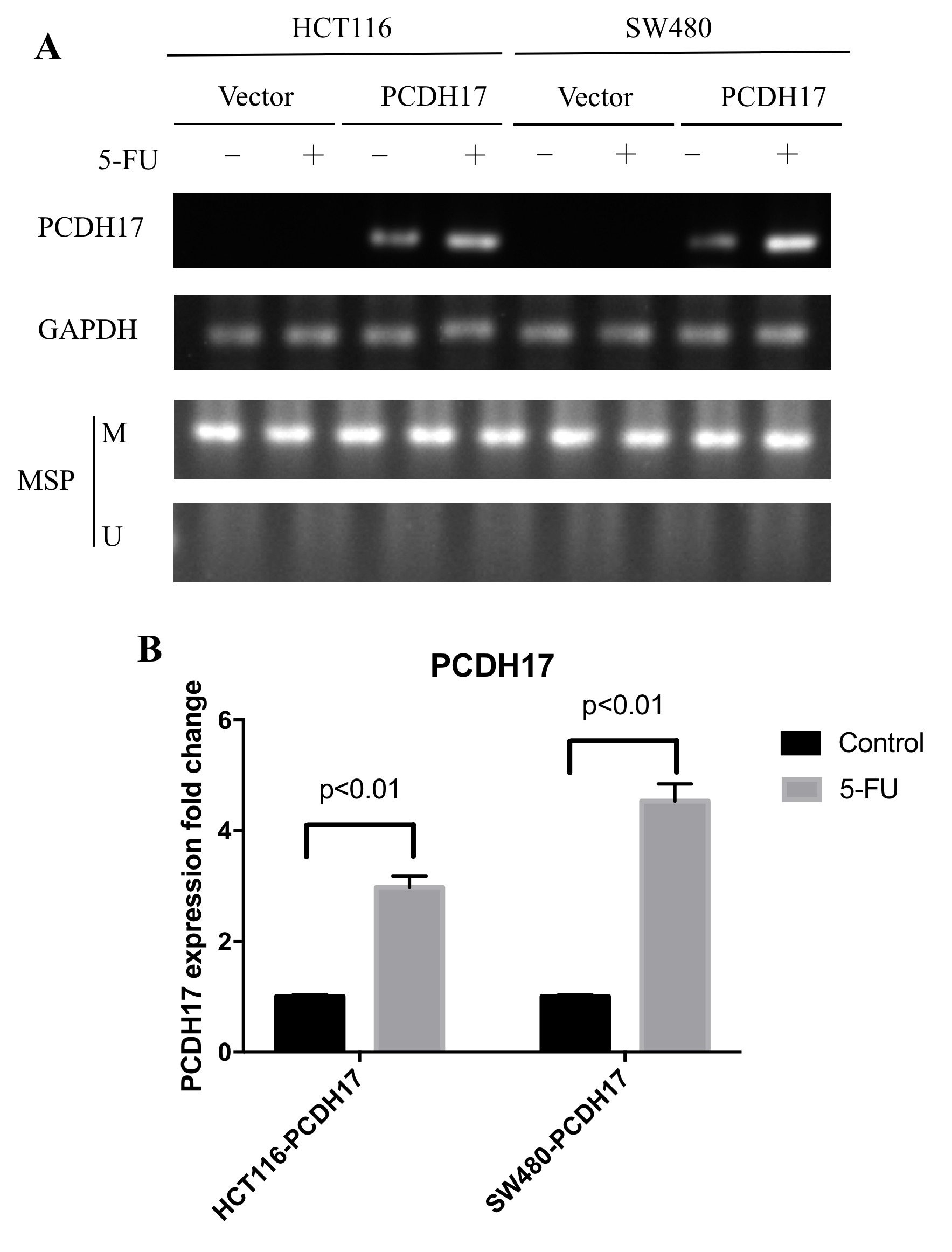


**Figure S2. The effect of 5-FU on PCDH17 expression.**

(**a**) The effect of 5-FU on PCDH17 expression was determined by PCR and methylation-specific PCR (MSP), with GAPDH as a control. M: methylated; U: unmethylated. Colorectal cancer cell lines, including HCT116/vector, HCT116/*PCDH17*, SW480/vector, and SW480/*PCDH17*, were used. 24 hours after treating with or without 20 μM 5-FU, total RNA was extracted using the Trizol reagent as described by the manufacture. The mRNA expression levels of the *PCDH17,* methylated *PCDH17,* and unmethylated *PCDH17* were determined by reverse-transcription PCR or methylation-specific PCR (MSP) with special primers respectively.

(**b**) mRNA expression level of PCDH17 is confirmed by qRT-PCR. Colorectal cancer cell lines HCT116/*PCDH17* and SW480/*PCDH17* were used. 24 hours after treating with or without 20 μM 5-FU, total RNA was extracted using the Trizol reagent as described by the manufacture. The mRNA expression levels of the *PCDH17* was determined by quantitative reverse-transcription PCR (qRT-PCR) with special primers. The transcription of the principal gene GAPDH was used as the internal control. Data represents means ± SEM from at least three independent experiments (p< 0.01)
